# The COVID-19 pandemic in Greenland, epidemic features and impact of early strict measures, March 2020 to June 2022

**DOI:** 10.2807/1560-7917.ES.2023.28.29.2200767

**Published:** 2023-07-20

**Authors:** Paneeraq Noahsen, Louise Lohmann Faber, Silvia Isidor, Jannik Fonager, Morten Rasmussen, Henrik L Hansen

**Affiliations:** 1 National Board of Health in Greenland, Nuuk, Greenland; 2 Ilisimatusarfik, University of Greenland, Nuuk, Greenland; 3 Aalborg University, Aalborg, Denmark; 4 Arctic Health Research Centre, Aalborg University Hospital, Aalborg, Denmark; 5 Virus Research and Development section, Virus and Microbiological Special Diagnostics, Statens Serum Institut, Copenhagen, Denmark

**Keywords:** COVID-19, SARS-CoV-2, Greenland, epidemiology

## Abstract

**Background:**

The COVID-19 pandemic was of major concern in Greenland. There was a high possibility of rapid transmission in settlements, and an increased risk of morbidity and mortality because of comorbidities in the population and limited access to specialised healthcare in remote areas.

**Aim:**

To describe the epidemiology of the COVID-19 pandemic in Greenland and evaluate the effects of a strict COVID-19 strategy until risk groups were immunised.

**Methods:**

We studied the epidemiology during March 2020 to June 2022. We describe the non-pharmaceutical interventions (NPIs), PCR-confirmed COVID-19 cases and vaccination coverage with data from the registries of the Greenlandic health authority.

**Results:**

We found 21,419 confirmed cases per 100,000 inhabitants (54% female, 46% male), 342 per 100,000 were hospitalised and 16 per 100,000 were admitted to the intensive care unit. The COVID-19 mortality rate was 39 per 100,000, all those affected were aged above 65 years. No excess overall mortality was observed. The vaccination coverage by June 2022 was 71.67 and 41% for one, two and three doses, respectively.

**Conclusion:**

SARS-CoV-2 circulation in Greenland was low, given strict restrictions until all eligible inhabitants had been offered immunisation. The main impact of the pandemic was from May 2021 onwards with increasing numbers of confirmed cases. This occurred after introduction of the vaccine programme, which may have had an influence on the severity of the associated morbidity and mortality experienced. Halting community transmission of SARS-CoV-2 with NPIs until the majority of the population had been immunised was a successful strategy in Greenland.

**Key public health message**

**What did you want to address in this study?**

We wanted to evaluate the effect of Greenland’s isolation and strict restrictions that were initiated in the first year of the COVID-19 pandemic on COVID-19 morbidity and mortality.

**What have we learnt from this study?**

We learned that delaying the community transmission of the SARS-CoV-2 through measures including lockdowns, strict control of travellers and restrictions on public gatherings until the majority of the population had been immunised resulted in low COVID-19 morbidity and mortality.

**What are the implications of your findings for public health?**

Being a remote island has advantages during a pandemic, as the import of the infectious disease can be kept at bay. Populations at risk for infection should be the focus of public health interventions to improve infection control and other preventive measures.

## Introduction

At the end of 2019, the Wuhan Municipal Health Commission reported an emergence of a severe respiratory disease. The causative virus was thereafter identified as the novel severe acute respiratory syndrome coronavirus 2 (SARS-CoV-2) [1,2]. This virus spread rapidly worldwide causing thousands of COVID-19 cases and deaths, and on 11 March 2020, the World Health Organization (WHO) proclaimed the pandemic [3]. Studies have shown increasing incidence and mortality of COVID-19 with increasing age [4], underlying health conditions [5] and sociodemographic risk factors, such as socioeconomic status, racial/ethnic minority status, environmental factors and household composition [6].

Greenland is a large island situated in the northern hemisphere between Canada and Iceland. The population of 56,367 [7] individuals, mostly Inuit, is spread across numerous settlements, from small villages to bigger towns and the capital Nuuk. The average life span is 69.5 years for men and 74.1 years for women. The majority of the population (57%) is aged between 25 to 64 years, while 9% of the population is 65 years or older. Despite improved living conditions in Greenland since the 1950’s, the prevalence of various infectious diseases such as tuberculosis and other upper and lower respiratory infections is still high, causing morbidity and mortality [8]. Moreover, there is inequality and inequity in health in Greenland. The population in the rural villages and in the capital Nuuk have substantially poorer access to the healthcare facilities compared with the population in other towns on the island [9]. The analysis of risk factors for infectious diseases has shown that being Inuit, living in overcrowded households and indoor smoking increases the risk of contracting otitis media with a hazard ratio of 5.56, 5.55 and 4.56, respectively [10]. Furthermore, the transition to Western diet and lifestyle has increased the prevalence of lifestyle related diseases such as obesity, cardiovascular diseases and diabetes [11]. 

The COVID-19 pandemic was thus of major concern in Greenland. The possibility of a rapid transmission in settlements was large, and there was a risk of high morbidity and mortality rates. In March 2020 when WHO proclaimed the pandemic, an advisory group called Strategic Corona Stab (STS) was established in Greenland, which provided the government with up-to-date situation reports and propositions on how to respond to the COVID-19 pandemic. Based on advice from the STS, the government of Greenland implemented a strict non-COVID-19 strategy, which included non-pharmaceutical interventions (NPIs), self-isolation of confirmed COVID-19 cases and voluntary quarantine of close contacts to suppress community spread of SARS-CoV-2. The aim was to shield the island from the pandemic until the Greenlandic population had been vaccinated. All suspected cases with COVID-19 and their close contacts were tested systematically for SARS-CoV-2 by PCR.

Here, we describe the epidemiology of the COVID-19 pandemic in Greenland and evaluate the effects of strict COVID-19 strategy that lasted until the immunisation of the risk groups was complete.

## Methods

### Study setting, population and period

Greenland is an island on the North American continent. The island is 2,166,086 m^2^ in size, of which 81% is covered by ice. Greenland is a self-governing territory in the Kingdom of Denmark. The capital of Greenland is Nuuk with a population of 19,604, and the larger towns are Sisimiut, Ilulissat, Aasiaat and Qaqortoq, with 5,436, 4,848, 3,069, 3,005 inhabitants respectively, all located on the west coast. 

The study population in this descriptive epidemiological study includes all 56,367 residents of Greenland (89.7% Inuit and 10.3% non-Inuit), as on 1 July 2020. The study period was from 1 March 2020 to 1 June 2022.

The Corona Secretariat was established by STS in March 2020 to provide manpower to communicate the NPIs, such as production of information campaigns, management of the Corona Hotline, contact tracing and systematic follow-up on all the COVID-19 cases and their close contacts.

### Case definitions

Confirmed COVID-19 cases are defined as patients who tested positive for severe acute respiratory syndrome coronavirus 2 (SARS-CoV-2) from nasopharyngeal or combined nasal and nasopharyngeal swabs analysed by PCR in clinical laboratories. Cases reported more than once within 4 weeks between the first and the second positive test were identified, and the latter removed from the registry. Clinically suspected cases are not included in our analysis because of a lack of available data.

A COVID-19-related death is defined as a death of a person with a laboratory-confirmed positive PCR SARS-CoV-2 test within 30 days from the first positive test. The COVID-19-related deaths are described in three categories: (i) COVID-19 as the primary cause of death, (ii) death because of underlying medical conditions accelerated by COVID-19 and (iii) death by other causes than COVID-19 in patients who tested positive for SARS-CoV-2 during the 30 days before death. International Classification of Diseases 10^th^ Revision (ICD-10) codes used for identifying COVID-19-related deaths were U07.1 ‘COVID-19, virus identified’ and U07.2 ‘COVID-19, virus not identified’ [12].

### Vaccinations

The vaccinations with mRNA vaccines against COVID-19 (Comirnaty (BNT162b2 mRNA, BioNTech-Pfizer) and Spikevax (mRNA-1273, Moderna)) started in January 2021 in Greenland. Because of the scarce number of available vaccines, older individuals (aged ≥ 65 years) and healthcare workers in healthcare facilities and nursing homes were prioritised initially. As the vaccine availability increased, vaccinations were offered to the remainder of the population (aged ≥ 12 years). ‘Fully vaccinated’ is defined as a person immunised two times with an mRNA COVID-19 vaccine.

Booster vaccinations were available from the last quarter of 2021 and from the beginning of 2022 in the more remote areas. Because of the geography and the infrastructure of Greenland, the vaccinations were administered starting from the capital and the larger towns and later followed smaller towns and villages.

### Data sources

Data on all positive cases including hospital admissions, COVID-19-related deaths and vaccination rates are from the National SARS-CoV-2 Registry at the National Board of Health, as all the COVID-19 cases and vaccinations are reported by the healthcare system to the National Board of Health. 

The surveillance system was robust throughout the pandemic in terms of the reporting of the confirmed cases, as the results from the laboratories were automatically generated and sent to the registry. 

Vaccinations administered in Denmark to citizens of Greenland are not reported to The National Board of Health in Greenland (number not known) and are therefore not included in the study.

The COVID-19-related deaths were identified with ICD-10 codes from death certificates. Death certificates with the cause of death registered as lower respiratory infection (ICD-10 codes J10-J18) or sepsis (ICD-10 codes A41, A418, A419) [12] from the last quarter of 2021 were identified and paired with the laboratory diagnostics before death.

Data on overall mortality in each quarter of 2018–21 plus the first two quarters of 2022 were obtained from Statistics Greenland. The mortality data in Statistics Greenland is based on data from The Danish Civil Registration System (CPR). All deaths in Greenland, as for the rest of the Kingdom of Denmark, are reported to The Danish Civil Registration System shortly after occurrence. The data are available online [7].

### Laboratory analysis

At the beginning of the pandemic, Greenland did not have laboratory capacity to analyse SARS-CoV-2. Nasopharyngeal swabs were air transported to Statens Serum Institut (SSI) in Copenhagen, Denmark for analyses. This caused a diagnostic delay of up to 1 week, depending on the geographical origin of the samples.

In April 2020, a PCR laboratory for SARS-CoV-2 testing was set up in Queen Ingrid’s Hospital in Nuuk. Thereafter, cartridge-based PCR test machines were eventually introduced in all four regional hospitals (located in towns with more than 3,000 inhabitants – Aasiaat, Ilulissat, Sisimiut and Qaqortoq) and in three health care centres in smaller and more remote towns with less than 3,000 inhabitants (Tasiilaq, Maniitsoq and Upernavik). SARS-CoV-2 antigen tests were available in health care centres from mid-2020 and distributed broadly by the end of 2021. All patients who tested positive for SARS-CoV-2 using rapid antigen tests in healthcare facilities were subsequently tested again for PCR analysis. The results of the rapid antigen tests taken in private homes were not reported to the Greenlandic health authority and were therefore not included in this study.

Nasopharyngeal or nasal swabs sent to SSI to analysis for SARS-CoV-2 were analysed using primers that target the E-gene on SARS-CoV-2 [13], where quantification cycle (Cq) values of 38 or less were defined as positive [14]. Positive samples were subsequently whole genome sequenced with Illumina sequencing technology, using a slightly modified ARTIC v3 amplicon sequencing panel (https://artic.network). After sequencing on either the NextSeq or NovaSeq platform, subvariants were called on subsequent consensus sequences containing < 3,000 ambiguous or missing sites using Pangolin (version 3.1.20) with the PangoLEARN assignment algorithm [15].

The Cepheid GeneXpert RT-PCR machines were introduced in the several healthcare facilities in Greenland and nasopharyngeal or nasal swabs were analysed locally for SARS-CoV-2 using the Cepheid Xpert Xpress SARS-CoV-2 assay.

### Statistical analysis

Data were processed and analysed using Excel (Microsoft Corp.) and the Statistical Package for the Social Sciences version 13.0 (SPSS Inc.). The total confirmed cases with COVID-19, hospitalisations and deaths COVID-19-related deaths represented as incidence per 100,000 inhabitants and percentage of the total number of cases. Age was given as median with a range of minimum and maximum. The mortality rate for 2018 and 2019 was calculated for each quarter in both years and compared with the mortality rates from the same quarters of 2020, 2021 and 2022, respectively. A chi-squared test was used for comparison of proportions. A p value of less than 0.05 was considered significant.

## Results

The first case in Greenland tested positive for SARS-CoV-2 on 13 March 2020. Within just over 2 weeks, 10 more cases were identified, all in Nuuk. Five of the first cases in this first COVID-19 wave were imported and six were secondary cases. Case 1 was tested positive for SARS-CoV-2 6 days after arrival to Greenland. Cases 2–5 were identified 13, 10, 6 and 8 days post-arrival, respectively. All close contacts were traced, urged to self-quarantine and were tested according to the national contact tracing guidelines. Cases 1, 2 and 3 lived alone and had had no close contacts post-arrival. Case 4 lived in a household with two other members and transmitted the virus to both, and also to two close contacts in another household. Case 5 lived with two household members and infected both with the virus.

**Figure 1:**
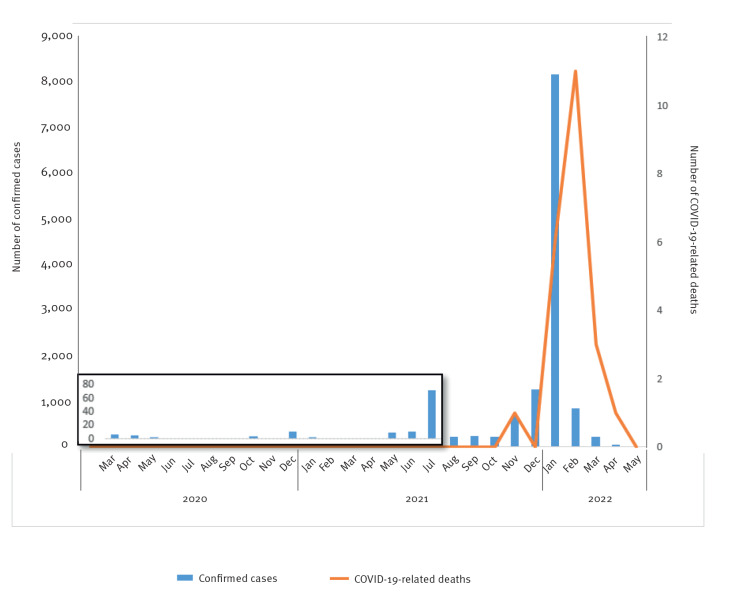
Confirmed COVID-19 cases (n = 12,073) and related deaths (n = 22), Greenland, 1 March 2020–1 June 2022

The confirmed COVID-19 cases were few and sporadic until the summer of 2021. In the following autumn and winter 2021, the virus spread quickly until case numbers peaked in January 2022 (Figure 1). A total number of confirmed cases in the study period (up to 1 June 2022) was 12,073, a cumulative incidence of 21,419 per 100,000 inhabitants.

The period from March 2020 through July 2021 is magnified because of the few numbers of confirmed COVID-19 cases.

Of the identified COVID-19 cases, 54% (n = 6,550) were female and 46% (n = 5,523) were male, and the median age was 30 years (range: 0–98). Among the COVID-19 cases, 60% (n = 7,232) were identified in Sermersooq municipality, where the capital Nuuk is located, 21% (n = 2,506) in Avannaata municipality, 9% (n = 1,046) in Qeqqata municipality, 6% (n= 733) in Qeqertalik municipality, and 5% (n = 556) in Kujalleq municipality.

In total, 1.6% of the confirmed cases were hospitalised (342/100,000 inhabitants) with a median age of 57 years (range: 0–96). Nine of the hospitalised patients were admitted in the intensive care unit (16/100,000 inhabitants), three of which were admitted because of other underlying health conditions. None of the patients needed respirator assistance.

### National non-COVID-19 strategy

On 18 March 2020, the capital Nuuk was locked down because of viral transmission in the community. The lockdown lasted until 27 April 2020 when it became clear that virus circulation was under control. Travel restrictions were implemented 20 March 2020 to prevent further import of SARS-CoV-2, and all international and domestic flights were cancelled. Greenland gradually re-opened during the summer and autumn 2020, but testing 5 days post-arrival was required for all international travellers. There were only 796 detected cases from beginning October 2020 to end October 2021, and 1,940 cases from November 2021 to December 2021. Stricter travel restrictions were reintroduced by the end of the 2021, until the last restrictions were lifted in the spring 2022 (Table 1).

**Table 1:** Overview of restrictions implemented during the COVID-19 pandemic, Greenland, 18 March 2020–18 May 2022

Date enacted	Local restrictions	International travel restrictions
18 Mar 2020	Lockdown in Nuuk. Public gathering limits outside of Nuuk (maximum 100 people). Visiting ban in hospitals and nursing homes. Travelling out of Nuuk prohibited.	A 14-day quarantine recommended for all international travellers.
20 Mar 2020	All domestic travelling cancelled. Schools for children between 6 and 16 years closed. Working from home recommended.	All international arrivals cancelled. A 14-day quarantine required from all international travellers.
27 Apr 2020	Public gathering restriction (maximum 100 people).	All international arrivals cancelled. No quarantine.
4 May 2020	Public gathering restriction (maximum 100 people).	Government-controlled travelling for necessary personnel. Traveller locater form, PCR test before departure, quarantine until a negative PCR test 5 days post-arrival required from all travellers.
15 Jun 2020	Public gathering restriction (maximum 100 people) but public gatherings with participants from other municipalities prohibited.	Limited opening for international travellers with maximum 600 arrivals per week. Traveller locater form, test before departure, quarantine until a negative test 5 days post-arrival required from all travellers.
21 Jul 2020	No new restrictions.	Unlimited opening for international travellers. Traveller locater form and test before departure required from all travellers.
29 Sep 2020	Mask mandate on domestic flights and in airports.	Unlimited opening for international travellers. Traveller locater form, test before departure, mask mandate and quarantine until a negative test 5 days post-arrival required from all travellers.
30 Dec 2020	No new restrictions.	All international arrivals cancelled.
5 Jan 2021	No new restrictions.	Government controlled travelling for necessary personnel. Traveller locater form, test before departure, quarantine until a negative test 5 days post-arrival required from all travellers.
20 Apr 2021	Mask mandate on domestic flights and in airports in place. Restrictions on public gatherings lifted.	No new restrictions. Government controlled travelling for necessary personnel. Traveller locater form, test before departure, quarantine until a negative test 5 days post-arrival required from all travellers.
3 May 2021	No new restrictions.	Limited opening for international travellers with maximum 600 arrivals per week. Traveller locater form, test before departure, quarantine until a negative test 5 days post-arrival required from all travellers.
28 May 2021	Travelling out of Nuuk prohibited until 1 June 2021. Mask mandate in all public places in Nuuk. Restaurants, cafés, pubs, and fitness centres closed in Nuuk.	No new restrictions.
1 Jun 2021	Mask mandate on domestic flights and in airports in place, all other restrictions lifted.	Quarantine and test post-arrival is no longer required for travellers who are vaccinated against COVID-19.
15 Jun 2021	Travelling out of Nuuk prohibited and mask mandate in all public places in Nuuk until 22 June 2021.	No new restrictions.
15 Jul 2021	Restrictions on public gathering (maximum 20 people) and mask mandate in public places in the whole country. Travelling out of two towns (Qaqortoq and Sisimiut) prohibited until 20 July 2021.	
28 Jul 2021	Restrictions for travellers from two regions in Greenland (Upernavik and Aasiaat including surrounding villages) requiring test before departure, quarantine until a negative test 5 days post-arrival from all travellers. Travellers vaccinated against COVID-19 not required testing or quarantine. Lifted 24 August 2021.	
10 Aug 2021	No restrictions in numbers for public gatherings. Full vaccination against COVID-19 or a negative PCR test for SARS-CoV-2 that is maximum 48 h old required upon entering any public places. Lifted 3 September 2021.	Limited opening for international travellers. All visitors must be vaccinated against COVID-19. Traveller locater form and test before departure required from all travellers.
23 Aug 2021	Restrictions for travellers in three regions (Upernavik, Aasiaat and Sisimiut including surrounding villages) requiring test before departure, quarantine until a negative test 5 days post-arrival from all travellers. Travellers vaccinated against COVID-19 not required testing or quarantine. Mask mandate in public places. Restrictions in public gathering (maximum 20 people) in above mentioned regions. Lifted 3 September 2021.	No new restrictions.
16 Sep 2021	Restrictions for travellers in two cities (Nuuk and Sisimiut) requiring test before departure, quarantine until a negative test 5 days post-arrival from all travellers. Travellers vaccinated against COVID-19 not required testing or quarantine. Mask mandate in public places. Full vaccination against COVID-19 or a negative PCR-test for SARS-CoV-2 that is maximum 48 h old required upon entering any public places in Nuuk and Sisimiut. Lifted 5 December 2021.	
20 Nov 2021	Restrictions for travellers in Upernavik and surrounding villages requiring test before departure, quarantine until a negative test 5 days post-arrival from all travellers. Travellers vaccinated against COVID-19 not required testing or quarantine. Mask mandate in public places. Full vaccination against COVID-19 or a negative PCR-test for SARS-CoV-2 that is maximum 48 h old required upon entering any public places in Upernavik. Lifted 5 December 2021.	
6 Dec 2021	Full vaccination against COVID-19 or a negative PCR-test for SARS-CoV-2 that is maximum 48 h old required upon entering any public places in the whole country. Grocery stores, post offices and other public offices excluded. Mask mandate in all cities and villages with active COVID-19 cases. Lifted 6 March 2022.	
25 Jan 2022	No new restrictions.	Limited opening for international travellers. All visitors must be vaccinated against COVID-19. Traveller locater form and test before departure no longer required.
18 May 2022	All local restrictions lifted.	All travel restriction lifted.

### Non-pharmaceutical interventions

Non-pharmaceutical interventions including social distancing, hand and respiratory hygiene, and limits on numbers of participants in public gatherings were introduced starting in March 2020 with mass communication campaigns as part of the strategy to prevent the SARS-CoV-2 transmission. As the pandemic progressed, a mask mandate was introduced, initially in the settlements with active outbreaks and eventually nationwide (Table 1).

### Vaccination coverage

The first COVID-19 vaccines were administered in Nuuk on 4 January 2021, followed by vaccinations in three towns (Ilulissat, Sisimiut and Qaqortoq) and subsequently in the smaller settlements. By May 2021, 25% of the population had been immunised with one vaccine dose, the majority of whom were aged above 60 years (Table 2). By June 2022, 71% of the Greenlandic population had received their first vaccination, while 67% and 41% had received their second and third doses, respectively.

**Table 2:** COVID-19 vaccination rates by age group, Greenland, May 2021 and June 2022 (n = 116,750 doses)

Age groups (years)	May 2021					June 2022						
	Total n	Dose 1		Dose 2		Total n	Dose 1		Dose 2		Dose 3	
		n	%	n	%		n	%	n	%	n	%
0–19	15,192	237	2	84	1	14,970	4,566	31	3,662	24	214	1
20–29	8,511	1,532	18	557	7	8,485	6,364	75	5,840	69	2,362	28
30–39	8,740	1,748	20	822	9	8,717	7,151	82	6,694	77	3,435	39
40–49	6,252	1,563	25	784	13	6,260	5,447	87	5,214	83	3,559	57
50–59	8,859	3,278	37	1,684	19	8,842	7,572	86	7,356	83	5,964	67
60–69	6,038	3,312	55	2,302	38	6,028	6,028	100	5,886	98	5,158	86
70–79	2,313	1,874	81	1,546	67	2,318	2,238	97	2,193	95	2,017	87
≥ 80	614	498	81	431	70	616	616	100	605	98	564	92
Total	56,519	14,042	25	8,201	15	56,416	39,982	71	37,450	66	23,273	41

Vaccinations administered in Denmark on citizens of Greenland were not included.

### Change in national strategy

On 10 January 2022, the national strategy of non-COVID-19 changed since the disease was no longer considered as a national health hazard. By this point, the number of suspected cases with COVID-19 and close contacts to confirmed cases had exceeded the testing capacity and the Corona Secretariat could no longer keep up with systematic follow-up and contact tracing of the SARS-CoV-2 positive cases. From 10 January, PCR tests were only accessible to patients who were at risk of serious illness with COVID-19, such as older people (aged ≥ 65 years), patients with underlying health conditions and patients admitted with upper and lower respiratory diseases to the healthcare centres or the regional hospitals. Nevertheless, much of the PCR testing continued in smaller towns during local outbreaks. The remainder of the population was encouraged to use rapid antigen tests at home and self-isolate in case of a positive test result.

### Mortality

Greenland registered 22 COVID-19-related deaths between March 2020 and June 2022. The first COVID-19-related death was reported in November 2021. As illustrated in Figure 1, the COVID-19-related deaths peaked in February 2022, when 11 individuals were registered as deceased within 30 days after a positive PCR test for SARS-CoV-2. The COVID-19 mortality rate was 39 per 100,000, yielding a case fatality rate of 0.18. The median age was 80 years (range: 66–92). As all fatalities were in individuals older than 65 years, the mortality rate in this age group was 441 per 100,000 inhabitants.

Fatalities were categorised in three groups. Eleven of the 22 COVID-19-related deaths had COVID-19 as the primary cause of death, and 2, 1 and 8 cases were vaccinated 0, 1 and 2 times, respectively. Eight cases died because of underlying medical conditions accelerated by COVID-19, with 3,1 and 4 cases vaccinated 0, 1 and 2 times, respectively. Three cases died of causes other than COVID-19, but had tested positive for SARS-CoV-2 30 days or less before their death; all three cases were vaccinated two times.

Death certificates from September 2021 onward, where the cause of death was registered as lower respiratory infection (ICD-10 codes J10-J18) or sepsis (ICD-10 code A41, A418, A419), were analysed. The analysis indicated an incomplete testing for SARS-CoV-2 in hospitals and healthcare centres in the smaller settlements. Of the 16 fatalities with pneumonia or sepsis, only five had been tested for SARS-CoV-2, and all were negative. The remaining 11 had not been tested for SARS-CoV-2 or other pathogens.

The overall mortality in the first 10 quarters of the pandemic (1 January 2020–30 June 2022) was compared with the overall mortality in Greenland during the same quarters in 2018 and 2019. The first quarter of 2020 was compared to the overall mortality in the first quarter of 2018 and 2019, the second quarter of 2020 to the mortality in the second quarter of 2018 and 2019, the third quarter of 2020 to the third quarter of 2018 and 2019, the fourth quarter of 2020 to the fourth quarter of 2018 and 2019. The four quarters of 2021 and the two first quarters of 2022 were compared with the corresponding quarters in 2018 and 2019 as described above. There were no statistically significant differences between observed deaths during the first 10 quarters of the pandemic compared with the expected deaths during the same quarters before the pandemic (p values between 0.82 and 1.21). There was a total of 1,297 recorded deaths during the first 2.5 years of pandemic, lower than the 1,308 expected deaths (p = 0.84).

### SARS-CoV-2 variants

**Figure 2:**
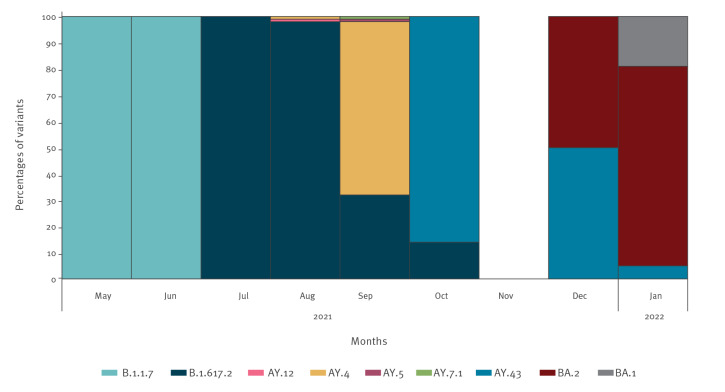
SARS-CoV-2 variants identified by whole genome sequencing, Greenland, 1 May 2021–31 January 2022 (n = 393)

Of the 12,073 positive SARS-CoV-2 tests, 393 were sequenced at Statens Serum Institut in Copenhagen, Denmark (Figure 2). In the second quarter of 2021, 9 of 22 positive tests were sequenced, and all were identified as the Alpha variant (Phylogenetic Assignment of Named Global Outbreak (Pango) lineage designation B.1.1.7). In the third quarter of 2021, the Delta variant (Pango lineage designation B.1.617.2) emerged and was the dominant variant, until the Omicron variant (Pango lineage designation B.1.1.529) was imported by winter holiday travellers. By the first quarter of 2022, the Omicron variant became the predominant variant (94% Omicron, 6% Delta).

Variants (Pango lineage designations): Alpha (B.1.1.7); Delta (B.1.617.2, AY.12, AY.4, AY.5, AY.7.1, AY.43); Omicron (BA.2, BA.1).

## Discussion

The confirmed COVID-19 cases in Greenland were few and sporadic until the summer of 2021, after which Greenland was impacted by the first wave in July 2021, followed by a larger wave from September 2021. The number of cases peaked in January 2022. Overall, the total number of COVID-19 cases in Greenland was high, considering the population size. However, both hospitalisation and death rate were low, possibly because of the strict NPI policy implemented from the beginning of the pandemic that likely shifted the peak until the majority of the population had been vaccinated. 

There were regional variations of the pandemic across the Arctic in the numbers of confirmed COVID-19 cases and in the durations of the waves. By 1 January 2022, the neighbouring Inuit regions in Alaska and the northern part of Canada had already experienced three waves [16]. From February 2020 to 1 January 2022, Inuit communities in northern Canada had recorded 3,649 confirmed cases per 100,000 inhabitants with 23 deaths per 100,000 inhabitants. Alaska was the Arctic region with most confirmed cases, with 22,102 COVID-19 cases per 100,000 inhabitants in all of Alaska. The number of COVID-19 cases in Greenland is very likely considerably higher than our study shows, as the PCR testing of suspected COVID-19 cases was halted on 10 January 2022, at a time when the need for testing exceeded the capacity for testing and analysing. In addition, test positivity rates varied widely both geographically and throughout the pandemic. Nevertheless, the cumulative incidence in the study period (up to 1 June 2022) in Greenland was 21,419 per 100,000 inhabitants which was much higher that the worldwide average of 7,277 confirmed cases per 100,000 inhabitants, registered 1 June 2022 by WHO [17]. In the Arctic regions, northern Russia was most severely impacted by the pandemic, with 232 deaths per 100,000 and case fatality rate of 2.8 [16]. The COVID-19 mortality rate in Greenland was 39 per 100,000 and the case fatality rate 0.18. Additionally, no excess overall mortality was observed during the COVID-19 pandemic in Greenland. 

Small population size and island status proved to be an asset during the pandemic, although it is evident that different approaches of island nations lead to diverse outcomes [18,19,20]. At the beginning of the pandemic, both Malta and Cyprus closed their borders, achieving lower SARS-CoV-2 positivity rates compared with Iceland, which kept borders open. In alignment with our findings, this study also showed that the transmission of SARS-CoV-2 and the mortality of COVID-19 could be controlled by implementing restrictive measures, e.g. closing of borders and restriction of movement. While Iceland’s strategy did not involve lockdown or closing of borders, there were restrictions on gatherings, testing and quarantine upon arrival from abroad, contact tracing, isolation and quarantine, and a mask mandate from August 2020 [20]. By June 2022, Iceland had confirmed 474 cases per 1,000 inhabitants, a mortality rate of 0.41 per 1,000 and case fatality rate of 0.09%. These numbers were much lower than the numbers observed in Greenland, and lower than the world average of 86 COVID-19-related deaths per 100,000 inhabitants and a case fatality rate of 1.17% during the same period [17]. New Zealand had similar strict non-COVID-19 policy and managed to eliminate the first minor waves with strict restrictions and lockdowns [18]. However, upon introduction of the Omicron variant, it was no longer possible for New Zealand to control virus spread. By June 2022, the number of confirmed COVID-19 cases in New Zealand reached 228 per 1,000, with 0.23 deaths per 1,000 inhabitants, resulting in case fatality ratio of 0.10% [19], which was still much lower than that observed in Greenland.

In spite of similar COVID-19 policies, the mortality rate is much higher in Greenland compared with island countries Iceland and New Zealand. It can be speculated that this is due to the double burden of non-communicable and infectious diseases seen in Greenland, as also seen in some lower-middle income countries. Greenland is in an epidemiological transition where the rates of infectious diseases are declining but still high and with an increasing burden of non-communicable diseases [21,22]. Other Inuit regions share the same challenges, and it is plausible that the double disease burden combined with limited resources and possibilities to halt the SARS-CoV-2 transmission in the communities led to high mortality rates in the Arctic.

As the restrictions were gradually lifted, it became increasingly difficult to keep COVID-19 out of Greenland. The first local outbreak occurred in May and June 2021 in Nuuk, when the more infectious Alpha variant [22] was imported and spread among workers in a larger construction site. Despite the limited number of confirmed COVID-19 cases, the local outbreak with the Alpha variant [23] caused the first admission in the intensive care unit. The SARS-CoV-2 Delta variant was introduced in the beginning of July 2021, and despite manageable numbers of cases, Greenland experienced an increase in numbers of hospitalisations during this period and the first COVID-19-related death occurred [24]. Notwithstanding the greater virulence of the Delta variant [25, 26], it was possible to eradicate the virus in the remote small communities in Greenland with strict NPIs. However, because of several superspreader events and continuous import of new cases from abroad, it was not possible to eliminate the virus in the larger towns before the arrival of the Omicron variant in December 2021.

With the import of the Omicron variant (Pango lineage designation BA.1 and BA.2), the number of infected individuals increased rapidly, and the strategy that was in use quickly became insufficient [27]. Because of the high number of COVID-19 cases, COVID-19-related hospitalisations and deaths peaked in the first quarter of 2022. Yet, the overall morbidity and mortality of the COVID-19 pandemic was limited in Greenland as the majority of the population was already vaccinated by then. The timing of the introduction of the Omicron variant may have contributed to the mild outcome of the COVID-19 pandemic in Greenland. It is well established that the Omicron variant has lower risk of hospital admission and lower risk of death compared with the Delta variant [28]. If the Omicron variant had not arrived and rapidly replaced the Delta variant, the outcome might have been very different.

The age distribution in the population may have contributed to the low morbidity and mortality observed [4]. The mean age of the population and the low number of people aged 80 years and older (1% of the population) is likely to have influenced the outcome. Even though the mortality in the total population is low, the mortality rate was high in those age 65 years and older (441 per 100,000). 

The relatively high vaccination coverage in Greenland is another important factor. By May 2021, the majority of the population aged 60 years or above had received both doses of the COVID-19 vaccine. By June 2022, 71% of the whole Greenlandic population had received their first doses, while 67% and 41% had received their second and third doses, respectively. Similar vaccination rates are seen in Alaska (73% first dose/65% second dose) [29]. A recent mathematical modelling study has shown that vaccinations have more than halved the potential global death toll because of COVID-19 [30]. The impact of the vaccination was highest in countries that prioritised their vaccination programmes before relaxing the NPIs, as we have seen in our study.

Our study has some limitations. Firstly, the PCR testing of every suspected case and close contacts to confirmed cases was stopped on 10 January 2022. From this point, only individuals at risk of high morbidity and mortality were offered PCR testing. Thus, the data do not include all COVID-19 cases, as the pandemic peaked in February 2022 according to the hospital admission data. Moreover, the results of the rapid antigen tests taken in private homes were not reported to the Greenlandic health authority and are therefore not included in this study. Secondly, the lack of laboratory capability to detect cases without any delay in the early part of the pandemic may have impacted the detection and reporting of COVID-19 cases. Thirdly, the fact that Greenland was ‘COVID-free’ for a substantial amount of time during the pandemic might have had an influence on the testing of patients with symptoms of upper respiratory diseases in the healthcare facilities. It can be speculated that the healthcare personal did not test patients with COVID-19 with the thought that the virus was kept at bay by the closed borders. The latter speculation is supported by the lack of testing for SARS-CoV-2 in patients with pneumonia in the smaller settlements and villages. Finally, the use of the timeframe of 30 days when reporting COVID-19-related fatalities may mean deaths due to COVID-19 that occurred at a later date were omitted from reporting. However, the same timeframe was used in Denmark [31]. The strength of this study is that we include all residents of Greenland and provide a detailed description of the NPIs and restrictions implemented in Greenland

## Conclusion

With the advantages of having an island status and with settlements spread far from each other along the vast coastline, Greenland kept SARS-CoV-2 at bay until nearly every person above 60 years had been fully vaccinated and many had received their booster shot. Consequently, the overall COVID-19 mortality was much lower in Greenland compared with other countries including the neighbouring Arctic regions, despite a high number of confirmed cases with SARS-CoV-2, considering the population size. We consider that halting the community transmission of the SARS-CoV-2 until the majority of the population had been immunised was a successful strategy for Greenland. As the previous epidemics and pandemics have nearly eliminated entire populations in small settlements in Greenland, the lessons learned from the management of the COVID-19 pandemic will undoubtably have implications on the future pandemic strategies in Greenland. 
